# Association of parental depression with educational outcomes in Indonesian children aged 6-12 years: A cross-sectional study

**DOI:** 10.51866/oa.931

**Published:** 2025-10-28

**Authors:** Rofingatul Mubasyiroh, Indri Yunita Suryaputri, Dini Rahma Bintari, Yurika Fauzia Wardhani, Eka Denis Machfutra, Ning Sulistiyowati, Sugiatmi Sugiatmi

**Affiliations:** 1 MPH, Research Center for Public Health and Nutrition, National Research and Innovation Agency Republic of Indonesia, Kawasan Sains Teknologi Dr. (H.C) Ir. H. Soekarno, Jl. Raya Jakarta-Bogor Km.46, Cibinong, Kabupaten Bogor, Jawa Barat, Indonesia. Email: rofingatul.mubasyiroh@brin.go.id; 2 M.Sc, Research Center for Public Health and Nutrition, National Research and Innovation Agency Republic of Indonesia, Kawasan Sains Teknologi Dr. (H.C) Ir. H. Soekarno, Jl. Raya Jakarta-Bogor Km.46, Cibinong, Kabupaten Bogor, Jawa Barat, Indonesia.; 3 M.Psi, PhD, Faculty of psychology, Universitas Indonesia, Jl. Lkr. Kampus Raya Jl. Prof. DR. R Slamet Iman Santoso, Pondok Cina, Kecamatan Beji, Kota Depok, Jawa Barat, Indonesia.; 4 M.Psi, Politeknik Kesehatan Kemenkes Surabaya, Jl. Pucang Jajar Tengah No.56, Kertajaya, Kec. Gubeng, Surabaya, Jawa Timur, Indonesia.; 5 MPH, Research Center for Public Health and Nutrition, National Research and Innovation Agency Republic of Indonesia, Kawasan Sains Teknologi Dr. (H.C) Ir. H. Soekarno, Jl. Raya Jakarta-Bogor Km.46, Cibinong, Kabupaten Bogor, Jawa Barat, Indonesia.; 6 MPH, Research Center for Public Health and Nutrition, National Research and Innovation Agency Republic of Indonesia, Kawasan Sains Teknologi Dr. (H.C) Ir. H. Soekarno, Jl. Raya Jakarta-Bogor Km.46, Cibinong, Kabupaten Bogor, Jawa Barat, Indonesia.; 7 PhD, Faculty of Medicine and Health, University of Muhammadiyah Jakarta, Jl. K.H. Ahmad Dahlan, Cireundeu, Kec. Ciputat Timur, Kota Tangerang Selatan, Banten, Indonesia.

**Keywords:** Parents, Depression, Student dropouts, Education, Child

## Abstract

**Introduction:**

Psychological factors affecting children have received less attention in relation to continued education, particularly among elementary school-aged children. This study aimed to investigate the association between parental depression and educational outcomes in children aged 6-12 years.

**Methods:**

This study was conducted using data from the Indonesian Family Life Survey. Children aged 6-12 years living with their parents were included in the study. The outcome variable was school dropout. The Center for Epidemiologic Studies Depression scale was used to assess parental depression as the independent variable. Multivariate logistic regression was performed with covariate adjustments to determine the association of parental depression with educational outcomes.

**Results:**

Approximately 8.7% of the children dropped out of school, with the prevalence being higher among the boys (11.6%) than among the girls (5.5%). After being adjusted for covariates, maternal depression was found to be associated with an increased risk of school dropout, particularly when it occurred during the children’s toddlerhood and childhood (odds ratio=1.97; 95% confidence interval=1.04-3.74; P=0.037). In contrast, paternal depression was not associated with an increased risk of school dropout.

**Conclusion:**

These findings suggest that maternal depression significantly influences the likelihood of school dropout among children.

## Introduction

Early childhood is marked by rapid physical growth and brain development. Multiple factors - genetic, biological, social and psychological - play a significant role in shaping early childhood development.^1,2^ Failure to nurture foundational skills, such as cognitive, social and emotional abilities, during this critical period may hinder a child’s capacity to learn and thrive in later stages of life.^3^ Preparing children adequately during early childhood is therefore essential for ensuring a nation’s long-term sustainability, progress and self-reliance. Thus, children’s education is often portrayed as the centre of attention in human resource development.

Education is a benchmark for human development indicators. Indonesia has stated the goals of education in the 1945 Constitution of Republic of Indonesia, namely, to develop the intellectual life of the nation and advance Indonesian education. Therefore, education for children is an essential part of the potential development process. Globally, according to UNICEF, more than 258 million children did not attend school in 2018, accounting for approximately 17% of the population of school-aged children (6-17 years of age).^4^ In Indonesia, as many as 40,823 children, or 0.17% of those aged 7-12 years in elementary schools, dropped out in 2022.^5^ Study findings in Aceh and Banten - two provinces in Indonesia with lower-than-average secondary school enrolment rates - suggested that high tuition fees accounted for the majority of dropouts, with behavioural issues cited by a subset of male dropouts.^6^ In addition to financial reasons, school dropout is also associated with psychological problems such as lack of interest and mental health problems.^7,8^

Psychological factors affecting children have received less attention in relation to continuing children’s education, particularly among those of elementary school age. Several studies have demonstrated that psychological variables, particularly maternal psychological conditions, influence children’s education, whether through dropouts, spelling, reading or math abilities.^9-11^ This study aimed to assess the effect of parents’ (both mother’s and father’s) psychological health, specifically depressive status, on the likelihood of elementary school-aged children dropping out of school.

## Methods

### Study design and participants

This research analysed data from the Indonesian Family Life Survey (IFLS), which are publicly available. The IFLS is a longitudinal survey that began in 1993 and was conducted in 1993, 1997, 2000, 2007 and 2014. The study was conducted in 13 selected provinces in Indonesia. IFLS respondents include people in a household selected in those provinces.^12^ The data used in this analysis were from the fourth and fifth IFLS, conducted in 2007 and 2014, respectively. The research population comprised children aged 6-12 years in 2014. Data of individual children in 2014 were matched with data from 2007, including data from their parents. Thus, this analysis used two-period longitudinal data on the same individual children and parents. Children living in the same house with their parents in 2007 and 2014 were included. Children who dropped out of school due to family economic reasons, did not attend school, had schools located too far from their homes or had no teachers available were excluded. There was a possibility of missing data due to changes in respondents over the two periods. Only children aged 6-12 years had all complete variables of data. The analysis began with 3637 children who met the inclusion criteria and lived with their parents and concluded with 2436 children with complete data on all household, parental and child variables.

### Variables

**Dependent variable**

The dependent variable was school dropout, defined as quitting school, failing or repeating a grade and temporarily dropping out (for at least 1 month). Dropout data were gathered from the IFLS-5, administered in 2014, when children reached school age. These data were collected as part of the Book 5 (Section DLA) questionnaire. A child was classified as having dropped out when any of the following applied: The answer was ‘no’ to the question, ‘Did [child’s name] graduate this level of schooling?’; ‘yes’ to ‘Has [child’s name] ever failed a grade at [...] school?’; or ‘yes’ to ‘Has [child’s name] ever left [...] and reentered?’.^12^

**Independent variables**

The primary independent variables in this research were maternal and paternal depressive conditions. Information on mothers’ and fathers’ depression was obtained from the IFLS-4 and IFLS-5, which were conducted in 2007 and 2014, respectively. The Center for Epidemiologic Studies Depression-10 (CES-D) from Book 3B, Section KP (Mental Health), was used to collect data on depressive symptoms. The CES-D 10 was validated in 1998 using confirmatory analysis, demonstrating that the instrument was valid and appropriate for the Indonesian cultural context.^13^ According to a systematic review and meta-analysis of 33 trials, the CES-D demonstrated consistent diagnostic accuracy for screening major depressive disorder (MDD), with pooled sensitivity and specificity of 0.8 or higher and an sROC AUC of 0.9 or higher in adults. Finally, its efficacy in screening for MDD suggested that it is a universally applicable depression screening tool in adults.^14^ The CES-D 10 showed a sensitivity of 0.81 and a specificity of 0.74, with a cutoff value of ≥10 in the general population aged 18-65 years in one of the systematic supporting studies conducted in Austria.^15^

The CES-D was applied in the IFLS in 2007 and 2014 and was administered to respondents aged 15 years or older. The CES-D used in the IFLS consisted of 10 questions regarding the frequency with which respondents experienced a mental health disorder in the preceding week. Each question contained four possible responses: 1) infrequently or never (1 day), 2) occasionally (1-2 days), 3) occasionally (3-4 days) and 4) most of the time (5-7 days). Each response was scored based on the scoring protocol of the CES-D. For example, for Questions 5 and 8, the highest score (score of 3) corresponded to Response Code 1 (rarely or none). For the remaining questions, the highest score (score of 3) corresponded to Response Code 4 (most of the time). Scores on each question were counted and categorised into a depressive condition when the scores were 10 or higher.^16^ This cut-off value was also employed in several other research that used the CES-D.^17,18^ The depressive status relative to the two assessment periods was categorised as follows: normal, depression in 2007 (when the child was in toddlerhood), depression in 2014 (when the child was in childhood) and depression during the two periods (when the child was in toddlerhood and childhood). In addition, the father’s and mother’s depression statuses were composited into parental depressive condition. It was classified as normal when neither parent had ever experienced depression and as depressive when at least one parent had experienced depression during their child’s toddlerhood or childhood.

**Covariate variables**

Covariate variables were obtained from data collected during toddlerhood and childhood. The covariates considered were as follows: child’s sex; history of infectious illnesses (measles, rubella, tuberculosis or diphtheria), polio, asthma, allergies, severe diarrhoea, epilepsy, neuropathy, psychiatric problems, diabetes, heart disease, leukaemia or cancer; child’s age; father’s age; mother’s and father’s occupations; mother’s and father’s educational levels; and child’s place of residence.

### Data analysis

Data analysis was conducted using both bivariate descriptive and multivariate methods. Bivariate descriptive analysis was used to illustrate the distribution and relationship of school dropout with participant characteristics as well as parental depression. Finally, multivariate logistic regression was conducted to determine the association between parental depression and school dropout. Odds ratios (ORs) were adjusted for the demographic characteristics, with the significance level set at P<0.05 and confidence interval (CI) at 95%.

## Results

Table 1 shows that there were more boys than girls among the participants. Almost half of the children (45.9%) had chronic diseases, had mothers who had low educational attainment and were unemployed and had fathers who had higher educational attainment and were employed. The majority (60.7%) lived in urban areas. Approximately 8.7% dropped out of school. Dropout was more common among the boys (11.6%). Low parental educational attainment was more significantly prevalent in rural areas (11.3%). The dropout rate was higher in the children whose fathers and mothers were unemployed, although the difference was not significant.

**Table 1:** Distribution of school dropout according to the participant characteristics.

	Overall		No dropout		School dropout		P-value
**Child characteristics**							
Total	2436	100.0	2225	91.3	211	8.7	
**Sex**							
Boy	1271	52.2	1124	88.4	147	11.6	
Girl	1165	47.8	1101	94.5	64	5.5	
Chronic illness							
No	1317	54.1	1194	90.7	123	9.3	
Yes	1119	45.9	1031	92.1	88	7.9	
**Parental characteristics**							
Mother’s age, year							
≤35	1139	46.8	1043	91.6	96	8.4	
36-45	1062	43.6	971	91.4	91	8.6	
46-55	226	9.3	203	89.8	23	10.2	
56-65	9	0.4	8	88.9	1	11.1	
**Father’s age, year**							0.242
≤35	549	22.5	512	93.3	37	6.7	
36-45	1293	53.1	1179	91.2	114	8.8	
46-55	509	20.9	458	90.0	51	10.0	
56-65	85	3.5	76	89.4	9	10.6	
**Mother’s educational attainment**							<0.001
Elementary school or below	782	32.1	674	86.2	108	13.8	
Middle school	618	25.4	561	90.8	57	9.2	
High school	743	30.5	705	94.9	38	5.1	
Bachelor’s degree or higher	293	12.0	285	97.3	8	2.7	
**Father’s educational attainment**							<0.001
Elementary school or below	775	31.8	656	84.6	119	15.4	
Middle school	465	19.1	423	91.0	42	9.0	
High school	855	35.1	810	94.7	45	5.3	
Bachelor’s degree or higher	341	14.0	336	98.5	5	1.5	
**Mother's employment status**							0.740
Working	1065	43.7	975	91.6	90	8.4	
Not working	1370	56.3	1249	91.2	121	8.8	
**Father's employment status**							0.740
Working	2244	92.1	2054	91.5	190	8.5	
Not working	192	7.9	171	89.1	21	10.9	
**Household characteristics**							
**Area of residence**							0.243
Rural area	957	39.3	849	88.7	108	11.3	
Urban area	1479	60.7	1376	93.0	103	7.0	

The depressive status of the mothers and fathers is shown in Table 2. Depression was slightly more prevalent among the mothers (29.3%) in both 2007 and 2014 than among the fathers (15.9%). Approximately 45.5% of the children had at least one parent who experienced depression during the two periods. A significant relationship (P=0.0021) was observed between maternal depression and school dropout among the children. Dropout occurred more frequently among the children who had a depressive mother during their toddlerhood and during both their toddlerhood and childhood over both periods. Conversely, the relationship between paternal depression and school dropout was not statistically significant. However, overall parental depressive experience was significantly associated with school dropout (P=0.009).

**Table 2:** Distribution of school dropout according to parental depression.

	Overall		No dropout		School dropout		P-value
	n	%	n	%	n	%	
**Child characteristics**							
All	2436	100.0	2225	91.3	211	8.7	
**Maternal depression**							0.021
Never	1708	70.7	1574	92.2	134	7.8	
Depression^1^	79	3.3	68	86.1	11	13.9	
Depression^2^	546	22.6	496	90.8	50	9.2	
Depression^3^	82	3.4	69	84.2	13	15.8	
**Paternal depression**							0.342
Never	1728	74.1	1588	91.9	140	8.1	
Depression^1^	75	3.2	65	86.7	10	13.3	
Depression^2^	458	19.6	417	91.1	41	8.9	
Depression^3^	72	3.1	64	88.9	8	11.1	
**Parental depression**							0.009
Never	1327	54.5	1230	92.7	97	7.3	
Had experience^4^	1109	45.5	995	89.7	114	10.3	

1 Parental depression during their child’s toddlerhood

2 Parental depression during their child’s childhood

3 Parental depression during their child’s toddlerhood and childhood

4 At least one parent experienced depression during their child’s toddlerhood or childhood.

In the multivariate logistic regression (Table 3), after adjustment for covariates, maternal depression was found to be associated with a higher risk of school dropout, particularly when the mother experienced depression during both their child’s toddlerhood and childhood (OR=1.97; 95% CI=1.04-3.74; P=0.037). In contrast, paternal depression was not associated with an increased risk of school dropout.

**Table 3:** Logistic regression of parental depression relative to school dropout.

Depressive condition	Adjusted OR (95% CI)	P-value
**Maternal depression**		
Never	1	
Depression^1^	1.78 (0.90–3.52)	0.099
Depression^2^	1.14 (0.81–1.62)	0.447
Depression^3^	1.97 (1.04–3.74)^*^	0.037
**Paternal depression**		
Never	1	
Depression^1^	1.48 (0.72–3.04)	0.280
Depression^2^	1.02 (0.70–1.49)	0.916
Depression^3^	1.35 (0.62–2.95)	0.448
**Parental depression**		
Never	1	
Had experience^4^	1.32 (0.98–1.77)	0.063

Adjustment for the child’s sex; history of chronic diseases; mother’s age; father’s age; mother’s and father’s occupations; mother’s and father’s educational attainment; and child’s residence

1 Parental depression during their child’s toddlerhood

2 Parental depression during their child’s childhood

3 Parental depression during both their child’s toddlerhood and childhood

4 At least one parent experienced depression during their child’s toddlerhood or childhood.

* Statistically significant at P<0.05

OR: odds ratio, CI: confidence interval

## Discussion

Analysis of the 2009 Susenas data showed that around 600,000 children aged 7-12 years did not attend school, including those who had dropped out. Data from 2011 indicated that the dropout rate for children aged 7-12 years was 0.67%.^19^ Conversely, a study in Ethiopia reported a dropout rate of 20.2% among children aged 6-7 years and 11.3% among those aged 7-8 years.^10^

The abovementioned results align with the 2009 Susenas data regarding the dropout percentage among elementary school-aged boys.^20^ A child whose mother has a low level of education is 20 times more likely to drop out of school than one whose mother is highly educated. Additionally, children who live in rural areas are twice as likely to be out of school, with the dropout rate for elementary school-aged children in these areas being two times higher. The same pattern is evident in the 2011 Susenas data.^19^ The higher dropout rate among children with unemployed parents compared to those with employed parents is also consistent with findings from the capital city, Jakarta.^21^

Maternal depression is associated with school dropout in children. This finding is in line with that of a study in Ethiopia, which showed that maternal depressive condition affected the occurrence of school dropout among children.^10^ The mechanisms through which maternal depression can impact a child’s education and well-being are complex and multifaceted. Some possible mechanisms include reduced emotional availability and responsiveness from the mother, leading to a lack of support and guidance for the child’s educational experience. Moreover, disruptions in the parent-child relationship affect the child’s sense of security and attachment. Increased stress and conflict within the family may create an unstable and challenging environment for the child. Thus, negative modelling of coping strategies and emotional regulation by the depressed mother can influence the child’s own emotional wellbeing and ability to cope with stress.^22^

In early childhood, children who are exposed to maternal depressive symptoms tend to exhibit negative behavioural outcomes, and lower levels of inhibitory control predict a stronger association between concurrent maternal depression and internalising and externalising behaviours.^23^ Vulnerability to maternal depressive symptoms during this critical period is negatively associated with cognitive outcomes in children, including academic performance. Evaluations have indicated small but consistent negative effects of early maternal depressive symptoms on later academic performance, with some studies indicating greater effects in boys.^24-26^

Other studies have concluded that certain levels of anxiety and internalising behaviours are significant risk factors for decreased emotional and cognitive engagement among elementary school students.^27^ Moreover, maternal depressive symptoms in late childhood and early adolescence (ages 8-13 years) are associated with greater substance abuse and delinquency among children, especially in boys, and depressive symptoms in girls.^28^ Behavioural problems are also recognised as risk factors for absenteeism and school dropout among children.^29^ A mother’s mental health condition, including depression, has distinct consequences on her child’s developmental outcomes.

Maternal depression is mediated by negative parenting practices that impact children’s behaviours,^30^ especially psychological aggression, unresponsiveness and reactivity.^31,32^ The consequences of this effect of depression on parenting can extend to the academic domain. For instance, depression can cause mothers to become unfocused and less prepared to engage in stimulating activities for their children, such as reading or playing.^33^ Another study showed that maternal depressive symptoms have the potential to initiate pathways of risk that could impair long-term academic success, not always seen in early years in elementary schools. Moreover, the association between maternal depressive symptoms and academic performance has been explained through indirect effects via cumulative parenting risk (e.g. chaos, hostility or low school involvement) in second grade and, consequently, lower levels of child functioning in fifth grade, which are associated with poorer academic performance in tenth grade.^34^

According to Indonesia’s traditional societal system, mothers are mostly responsible for childcare. They oversee meeting their child’s basic emotional and physical requirements, including eating, developing emotional bonds and assisting with learning. As the family’s primary provider and decision-maker, fathers are in a more advantageous position.^35^ Indeed, it has been demonstrated that father participation in parenting positively affects children’s growth. Increased cognitive ability in children is also linked to fathers’ positive participation. Because of its link to higher levels of socioemotional competence, father engagement also affects how well children regulate their emotions. Fewer problematic behaviours and more sophisticated math abilities are linked to greater father engagement in disciplining.^36^

Our study did not examine the moderating effect of maternal education on the relationship between maternal depression and child achievement. However, other studies have identified differing influences of depression among mothers with high versus low levels of education. Depression among mothers with low educational attainment has a significant impact, while depression among those with higher educational attainment does not tend to have a similar effect on children’s educational achievement. This difference is believed to be related to mothers’ socioeconomic status.^11^

Through the Ministry of Research, Technology and Higher Education of Indonesia, Indonesia launched the Gerakan Sekolah Sehat (Healthy School Movement) programme, one of the focuses of which is mental health. This programme is implemented at all levels of education, including elementary school. The implementation of the mental health programme in schools can take the form of activities: socialisation relative to mental health (which also involves parents); implementation of joint prayers before and after learning; increasing the understanding and capacity of educators related to mental health; and implementation of mental health screening among students through coordination and cooperation with the health centre, which can be assisted or known by parents.^37,38^ This programme is excellent, as it allows parents to actively participate in promoting their children’s mental health. It would be even more impactful if the programme evolved into an intervention pathway that provides support for parents experiencing mental disorders. Regarding mental health support for parents, there is one programme in the United Kingdom that could be emulated. Place2Be is a charity programme committed to improving the mental health of children in partner schools. This programme also targets parents, in addition to children. Every parent has access to mental health services in the form of online parenting courses and childcare advice. If a family in a partner school is identified as needing additional support, they may receive a mental health assessment. Place2Be uses this assessment to ascertain if it can offer more focused and beneficial interventions.^39,40^

The findings of this study highlight the crucial role of maternal mental health in a child’s educational success. A strong commitment from all stakeholders is necessary to support children in completing their education. One alternative form of support that could be proposed is the expansion of access to quality childcare centres. These centres can serve as safe, nurturing environments for children, especially when mothers are experiencing mental health challenges.

### Strengths and limitations

One of our study’s strengths is the availability of complete paternal and maternal data, particularly on mental health issues. We also avoided potential bias by only selecting children living with their parents. However, our study also has some limitations, including the lack of complete covariate variables, such as stressful life events, parental substance use, nutritional status, parenting patterns and the number of siblings. Nevertheless, we used a validated depression assessment instrument and included only children who lived with their parents to maintain consistency in the analysis.

## Conclusion

Our findings indicate that maternal depression, both during children’s toddlerhood and childhood, has a significant association with school dropout in children. This study only presents an overview of the association, not causality. Further research is needed to explore the mechanisms through which maternal depression contributes to school dropout. However, improving literacy about parental mental health and parenting practices can help bridge the gap to enhance children’s educational outcomes.

## Data sharing statement

The data used in the analysis are from a public domain (IFLS), conducted by a reputable survey institution that adheres to established research ethics. The data can be downloaded from the following link: https://www.rand.org/well-being/social-and-behavioral-policy/data/FLS/IFLS/access.html.

**How does this paper make a difference in general practice?**

- This study presents evidence-based support on parental mental health and children’s academic achievement.
- School dropout is more prevalent among children whose parents experience depression. Maternal depression, in particular, is associated with an increased risk of school dropout.
- These findings highlight the importance of addressing children’s education through consideration of the psychosocial conditions surrounding them, especially those related to their parents.
